# Chemical, biological and *in silico* assessment of date (*P. dactylifera* L.) fruits grown in Ha’il region

**DOI:** 10.3389/fchem.2023.1138057

**Published:** 2023-03-03

**Authors:** Abdulmohsen Khalaf Dhahi Alsukaibi, Khalaf M. Alenezi, Ashanul Haque, Irfan Ahmad, Mohd Saeed, Mahima Verma, Irfan Ahmad Ansari, Ming-Fa Hsieh

**Affiliations:** ^1^ Department of Chemistry, College of Science, University of Ha’il, Hail, Saudi Arabia; ^2^ Department of Clinical Laboratory Science, College of Applied Medical Sciences, King Khalid University, Abha, Saudi Arabia; ^3^ Department of Biology, College of Science, University of Ha’il, Hail, Saudi Arabia; ^4^ Department of Biosciences, Integral University, Lucknow, India; ^5^ Department of Biomedical Engineering, Chung Yuan Christian University, Taoyuan City, Taiwan

**Keywords:** date fruits, extraction, GC-MS, Ha’il region, molecular docking, molecular dynamics, *P. dactylifera* L.

## Abstract

**Background:** Dates palm (*Phoenix dactylifera* L.) fruits are among the most widely used fruits in the Middle East and African nations. Numerous researchers confirmed the presence of phytochemicals in *P. dactylifera* L. fruit and its by-products with broad-ranging biological activities.

**Objectives:** In the present work, phytochemical and biological assessments of two different cultivars of date fruit (*Shishi* **M1** and *Majdool* **M2** grown in the Ha’il region of Saudi Arabia) have been carried out.

**Methods:** Date fruits were extracted and analyzed by gas chromatography-mass spectrometry (GS-MS),liquid chromatography-mass spectrometry (LC-MS) and Fourier-transform infrared spectroscopy (FT-IR)techniques. The lyophilized methanolic extracts were analyzed for their *in-vitro* antiproliferative andcytotoxicity against colon cancer (HCT116) cell line. To identify the possible constituents responsible for the bioactivity, *in-silico* molecular docking and molecular dynamics (MD) simulation studies were carried out.

**Results:** Both cultivars exhibited *in-vitro* anticancer activity (IC_50_ = 591.3 μg/mL and 449.9 μg/mL for **M1** and **M2**, respectively) against colon cancer HCT-116 cells. The computational analysis results indicated procyanidin B2 and luteolin-7-O-rutinoside as the active constituents.

**Conclusion:** Based on these results, we conclude that these cultivars could be a valuable source for developing health promoter phytochemicals, leading to the development of the Ha’il region, Saudi Arabia.

## Introduction

Natural products (NPs) play an instrumental role in drug design and remain an inspiration for discovering new drug candidates (Newman and Cragg, 2016). Being the largest source of new pharmacophores, 60%–70% of drugs used today are based directly or indirectly on NPs (Atanasov et al., 2021). Indeed, enormous diversity in their chemical structure, broad-ranging bioactivity, low toxicity, and ability to bind with different proteins (targets) gives natural compounds an edge over synthetic ones (Ali et al., 2010). In the quest for new drug candidates, research is being carried out on the extraction, isolation, and identification of bioactive compounds found in plants, animals and microbes (Altemimi et al., 2017). Among a large pool of NPs, the dates palm (*Phoenix dactylifera* L.), a member of the Asteraceae family, has garnered an immense interest (Al-Alawi et al., 2017; Maqsood et al., 2020). Date fruits of the date palm tree (*P. dactylifera* L.) is one of the most consumed fruits worldwide, especially in the Middle East and Asian countries (Complexity, 2022). Apart from their high nutritional and commercial values (Mia et al., 2020), date fruits and their by-products have also attracted researchers due to their potential health benefits (Maqsood et al., 2020). Their antibacterial, antifungal, antiviral, antidiabetic, anticancer, anti-inflammatory, antioxidant, antiangiogenic and other protective effects along with negligible side effects, are particularly interesting (Vayalil, 2002; Maqsood et al., 2020). It has been demonstrated that date fruits are rich in carbohydrates, protein, fibres, minerals, vitamins, phenolic acids, flavonoids, and other phytochemicals responsible for bioactivities. The chemical composition depends on various factors, including the type of cultivar, geographical location, irrigation method, ripening stage, processing time, extracting solvents, etc. (Borochov-Neori et al., 2013) Based on this knowledge, various groups investigated the chemical composition of date fruits and seeds native to different regions (Vayalil, 2012; Al-Alawi et al., 2017; Mia et al., 2020; Echegaray et al., 2021; Ibrahim et al., 2021). The group led by Aviram (Borochov-Neori et al., 2013) has conducted studies on the chemical and biological analysis of several varieties of date fruits (Maqsood et al., 2020). They, along with others, confirmed that the phytoconstituents and bioactivity of the date fruits are the function of the parameters mentioned above. For example, a pilot study showed that *Medjool* or *Hallawi* varieties of date fruits vary in phenolics, catechins and quercetin derivative content and antioxidant effect (Rock et al., 2009). The same group reported anti-atherogenic properties of acetone extracts of *Hallawi* in addition to the eight other variants (Borochov-Neori et al., 2013). As per the group, phenolic compounds exerted anti-atherogenic properties via low-density lipoprotein (LDL) oxidation and serum-mediated cholesterol efflux. On the other hand, alcoholic extract of the Tunisian variety was found to inhibit α-glucosidase and α-amylase enzymes with low IC_50_ values (El Abed et al., 2017). Zhang et al. (2013) performed an extensive chemical and biochemical profiling of *Ajwa* date fruits. They identified several new compounds such as bis (2-ethylhexyl) terephthalate and bis (2-ethylheptyl) phthalate) in addition to glycoside, terpenoids, triglyceride, phthalates, etc. They noted that the aqueous and organic extract exerts dose-dependent antioxidant and anti-inflammatory effects.

In Saudi Arabia, more than 400 varieties of date fruit are cultivated, which vary in appearance, nutrition, and nutritional value (Zhang et al., 2017). Several researchers reported that the varieties such as *Barni*, *Khalas*, and *Ajwa* show unique biological activities (Eid et al., 2013; Assirey, 2015; Hamad et al., 2015). Recently, Amir and co-workers (Alghamdi et al., 2018) studied the nutritional value of several varieties of date fruits found in Ha’il province; however, the biological activity of the date fruits remains unclear. Prompted by this, we carried out extraction, characterization, *in-vitro* antiproliferative and cytotoxicity assay of two varieties of date fruits (*Shishi* **M1** and *Majdool* **M2**) grown in the Ha’il region of Saudi Arabia). Then, to further identify the possible potential constituent present, ligand-based virtual screening was performed. This is followed by molecular dynamics simulation studies to identify the stability of promising compounds with possible receptor.

## Materials and methods

### General

All solvents used for isolation and purification were of ACS reagent grade (Sigma-Aldrich Chemical Co., St. Louis, MO, United States). Lyophilization was carried out on BenchTop Manifold Freeze Dryer (MILLROCK, United States) for 24 h at a condenser temperature of −45°C equipped with Edward pump. Attenuated-total-reflectance IR spectra were recorded on pure samples on diamond using a Shimadzu IRSpirit-T spectrometer.

### Sample collection, extraction, and sample preparation

Two different varieties of date fruits (*Tamr* stage, Supplementary Table S1) grown in Ha’il province were collected from the local market. Authors (AKDA and KMA) and local farmers authenticated the samples, and a voucher specimen was deposited. The samples were stored and kept in a −20°C freezer. First, three pieces of date fruits from each variant (Supplementary Table S1) were pitted to remove seeds and cut into small pieces. Then, cold extraction was performed by shaking and mixing fruit materials in methanol (MeOH) overnight at room temperature, followed by filtration. The residue was further extracted twice with MeOH for 1 h. Finally, the extracts were combined and concentrated using rotatory evaporation at room temperature. The resulting viscous honey-like liquid was lyophilized to afford light yellow water-soluble powder and stored at −20°C till further analysis.

### Chromatographic studies

Date fruit extracts were analyzed by LC-MS system using a reverse phase C_18_ column (Accucore, 150 × 4.6, 2.6 μm). The LC-MS system comprised a Waters Alliance 2695 HPLC pump, an autosampler, a vacuum degasser, and a column compartment attached to a XEVO-TQD detector with electrospray ionization (ESI). The following gradient of solvents were used: acetonitrile (mobile phase A) and 5 mM acetic acid (mobile phase B); ratio of A to B, 0–1 min, 5:95; 1–10 min, 5:95 to 30:70; 10–16 min, 30:70 to 60:40; 16–24 min, 60:40 to 80:20; 24–32 min, 80:20; 32–40 min, 5:95. In all cases, the columns were reequilibrated between injections with the equivalent of 5 mL of the mobile phase. During the full scan by MS/MS, mass acquisition was set from 150 to 2000 Da. This method utilized ESI-LC/MS/MS operating in MRM mode. The ESI settings were the following capillary voltage, 3.5 kV; cone voltage, 40 V; the flow of desolvation gas (Argon gas), 650 L/h; flow of cone gas, 30 L/h. Gas Chromatography-Mass Spectrometry (GC-MS) analysis was carried out using Agilent 8890/5977B Series (Agilent 5977B EI/CI MSD) spectrometer. The segments were recognized by examination of their delay times and mass spectra with those of the NIST 11 mass spectral database.

### Cell culture and maintenance

The human colon cancer cell HCT116 was acquired from American Type Culture Collection (ATCC). McCoy’s 5 A media supplemented with 10% v/v Fetal Bovine Serum (FBS), and 1% antibiotic-antimycotic solution (1 mL contains 10,000 U Penicillin, 10 mg Streptomycin and 25 µg Amphotericin B) was used to grow and maintain HCT116 cells. A humidified environment constituted the standard conditions for cell culture at 37°C with 5% CO_2_.

### Cell viability assay

To determine the cytotoxicity of **M1** and **M2** extracts on colon cancer HCT-116 cell line, MTT assay was used. In a 96-well plate, the cells (5 × 10^3^ cells/well) were cultured for 24 h. The cells were treated with **M1** and **M2** at varied concentrations (100, 500, 1,000, and 5,000 μg/mL) for 24 h, respectively. Each well received 10 µL of MTT solution (5 mg/mL) and was subjected to further 3 h incubation at 37°C. In order to dissolve the purple formazan crystals, 100 µL of dimethyl sulfoxide (DMSO) was added to each well. A microplate reader measured absorbance at 570 nm (Bio-Rad, United States). The cell viability was expressed as a percentage (%) over the untreated control. For calculating the IC_50_ value, GraphPad Prism Professional software was used.

### Morphological analysis

The effects of **M1** and **M2** extract treatment on the morphology of HCT-116 cells were investigated using a phase contrast microscope. Briefly, HCT-116 cells (5 × 10^3^) were cultivated in a 96-well plate before **M1** and **M2** (100, 500, 1,000, and 5,000 μg/mL) treatment. The alteration in morphology of **M1** and **M2**-treated cells was then examined using a phase contrast microscope (Labomed, United States).

### Trypan blue exclusion assay

Trypan blue dye exclusion assay was performed further to confirm the **M1** and **M2**-mediated cytotoxicity in HCT-116 cells. A hemocytometer and a microscope were used to count the cells (5 × 10^4^) after they had been co-cultured with and without **M1** and **M2** (100, 500, 1,000, and 5,000 μg/mL), respectively for 24 h. The proportion of dead cells in each treatment set from studies done in triplicates was used to express the results.

### Lactate dehydrogenase release assay

In accordance with the manufacturer’s instructions, the lactate dehydrogenase (LDH) release assay kit was used to measure the level of cellular cytotoxicity. First, **M1** and **M2** were applied to the HCT-116 cells using a 96-well plate at different doses (100, 500, 1,000, and 5,000 μg/mL) for 24 h. LDH release kit was then used to detect released LDH in both the **M1** and **M2**—treated HCT-116 cells in the incubation medium.

### Computational details

Computational calculations were carried out on a Dell workstation (Galax GeForce GTX 1660 Ti) equipped with 8-core processors, 64 GB Ram, and NVIDIA graphics card.

### Receptor and ligands preparation

The co-crystal structure of Bcl-2 complex (PDB ID: 5JSN) was selected for virtual screening and molecular dynamics studies. In the crystallographic structure of this complex, there is a gap at position 33–86, which was fixed by homology modelling using the Swiss modeller tool. Based on literature reports (Mia et al., 2020), a total of ninety-four (*n* = 94) phytoconstituents of different chemical classes (Supplementary Table S2) were selected. Chemical structures (.sdf format) of the compounds were retrieved from the NCBI PubChem database (Wang et al., 2009). The downloaded files were converted to .pdb format using the Open Babel software. The ligand files were prepared using AutoDock Tools 1.5.7 (the Scripps Research Institute, La Jolla, CA, United States) software and finally written as .pdbqt file format for docking studies (Ahamad et al., 2021a).

### Active site prediction

The Bcl-2 protein (PDB ID: 5JSN) was given as input to identify the active site, which gives significant insight into recognizing surface structural pockets, shape and volume of every pocket, internal cavities of protein and surface areas. Next, the active site and the interactive residues were selected using PDBsum and CASTp online tools (Laskowski et al., 2018; Tian et al., 2018). The ligands were prepared using AutoDock Tools (ADT), and saved in pdbqt format (Trott and Olson, 2010).

### Protein preparation and grid generation

The 3D structure of Bcl-2 was prepared using the ADT protein preparation wizard. The polar and missing hydrogen atoms were added, while water molecules and hetero-atoms were deleted (Forli et al., 2016). Energy minimization was performed with a default constraint of 0.3 Å root mean square (RMS) and charges were assigned. After protein preparation, clean structure was saved as pdbqt file. Grid box (84 Å × 82 Å × 84 Å) was generated around the centroid of compounds with assigned X, Y, and Z axis.

### Virtual screening and binding affinity calculation

To identify the potential compounds found in *P. dactylifera* L., a dataset of ninety-four compounds was utilized for the virtual screening. The pdbqt files were provided as input and screened against Bcl-2 (Duffy and Avery, 2012). Top two compounds (ranked based on the binding energy scores and the docking poses) were selected for further studies (Trott and Olson, 2010; Forli et al., 2016). The compounds with favourable binding poses were identified with the help of the lowest free energy (ΔG), defined using the equation as follows,

$$∆G=∆G_{complex}\;–\;\left(∆G_{enzyme}+∆G_{ligand}\right)$$

Where (∆G_complex_), (∆G_receptor_), and (∆G_ligand_) are the average values of Gibbs free energy for the complex, receptor, and ligand, respectively. The stability of the docked complex between the receptor-ligand exhibits more negative scores, revealing the high potency of the inhibitor. All the other docking parameters were kept default, and the docked complexes final visualization was performed using PyMOL tool (DeLano, 2002). The active pocket of Bcl-2 and docked pose of the top-ranked compounds were compared to find interactive orientations.

### Molecular dynamics (MD) simulation

MD simulations were performed for the best-docked complexes with maximum binding affinity scores using GROningen MAchine for Chemical Simulations (GROMACS) *v*ersion 5.18.3. Package (Abraham et al., 2015). The topology of Bcl-2 was generated using GROMOS9643a1 force field (Van Der Spoel et al., 2005). Due to the lack of suitable force field parameters for a drug-like molecule in the GROMACS software, the PRODRG server was used for the generation of molecular topologies and coordinate files (Schüttelkopf and Van Aalten, 2004). All the systems were solvated using a simple point charge model (SPC/E) in a cubic box. To neutralize the system 0.15 M counter ions (Na^+^ and Cl^−^) were added. The energy minimization of all the neutralized systems was performed using the steepest descent and conjugate gradients (50,000 steps for each). The constant number of particles, volume, and temperature (NVT) ensemble and constant number of particles, pressure, and temperature (NPT) ensemble were run for system equilibration (Ahamad et al., 2021b). Steepest descent followed by conjugate gradient algorithms was utilized on enzyme-ligand complexes. The NVT ensemble was employed at a constant temperature of 300 K and a constant pressure of 1bar. The SHAKE algorithm was used to confine the H atoms at their equilibrium distances and periodic boundary conditions. Moreover, the Particle Mesh Ewald (PME) method defines long-range electrostatic forces (Abraham et al., 2015). The cut-offs for van der Waals and columbic interactions were set as 1.0 nm. LINC algorithm was used to constrain the bonds and angles. Using the NPT ensemble, production runs were performed for 500 ns, with time integration. The energy, velocity, and trajectory were updated at the time interval of 10 ps The analysis is performed by using Cα-atom deviations of the protein calculated using root mean square deviations (RMSD). The relative fluctuations of each amino acid were defined with root mean square fluctuations (RMSF). To measure the compactness of a given molecule radius of gyrations (Rg) is implemented, and the solvent accessible surface area (SASA) was employed to know the electrostatic contributions of molecular solvation (Ahamad et al., 2018; Ahamad et al., 2019).

## Results

### Extraction and characterization of *Shishi* (M1) and *Majdool* (M2) fruits

Aqueous, organic or mixture solvents can extract a natural product, depending upon the analyte of interest (Nematallah et al., 2018). In the past, it has been demonstrated that when date fruits are extracted with an organic solvent, it yields bioactive compounds able to inhibit colon, liver and cervical cancerous cell lines *in vitro* (Mansour et al., 2011; Ravi, 2017). Especially, using a polar solvent such as methanol allows the extraction of various components (MHM et al., 2015). It was found that the alcoholic extract of the date fruits effectively inhibits α-glucosidase and α-amylase enzymes with low IC_50_ values in both *in-vitro* and *in-vivo* (El Abed et al., 2017). In 2016, Khan et al. (2016) demonstrated that the methanolic extract of *Ajwa* Date (Saudi origin) inhibits breast cancer (MCF-7) cell lines *via* cell cycle arrest and apoptosis. Motivated by this, we selected methanol as the extracting solvent in this study too. Cold extraction of finely cut **M1** (36.11 gm) and **M2** (46.22 gm) followed by concentration and lyophilization yielded **M1** and **M2** as a light-yellow water-soluble powder (9.1 and 15 gm of **M1** and **M2**, respectively). To identify the extract components, chromatographic (LC-MS & GC-MS) and spectroscopic (FT-IR) techniques (Supplementary Figures S1, S2) were employed.

**FIGURE 1:**
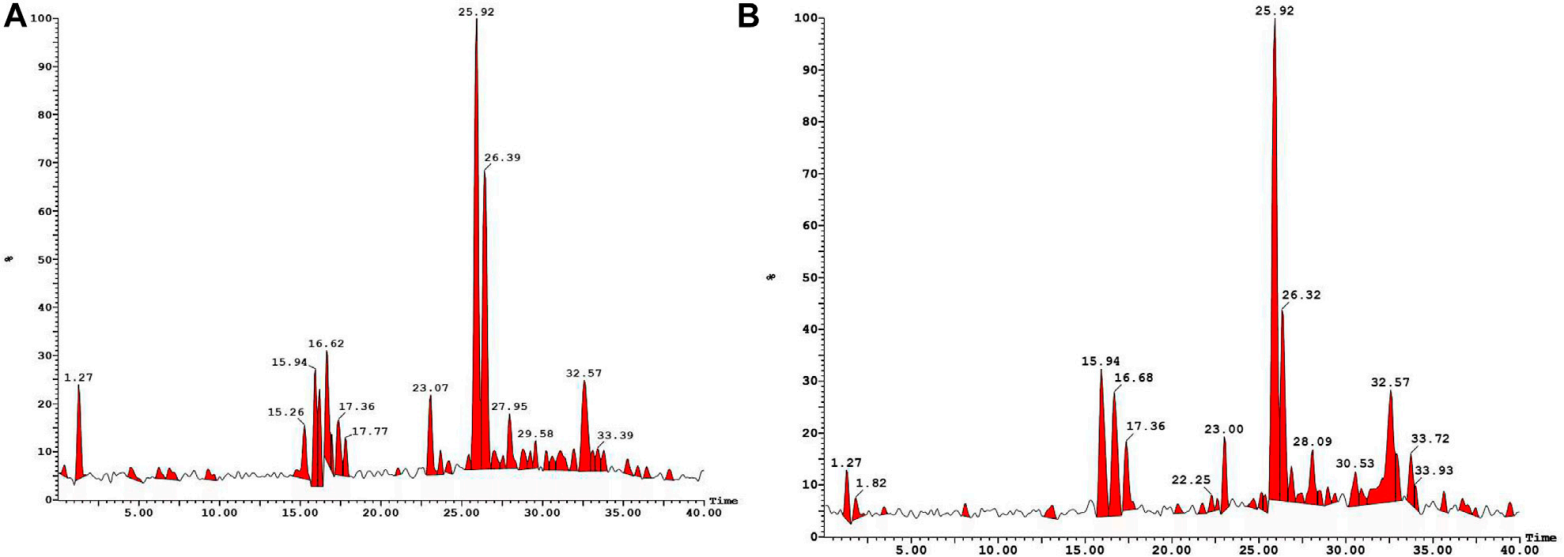
LC-MS (negative mode) traces of methanolic extracts of M1 **(A)** and M2 **(B)**.

Lyophilized products were analyzed by LC-MS (negative mode) using gradient mobile phase as it allows easy detection of phenolic acids & flavonoids as they contain acidic hydroxy group. It has been reported that the methanolic extract contains phenolic acids, flavonoid diglucosides, monoglucosides, acylated monoglucosides, free aglycones, lipids and others when analyzed under similar conditions (Farag et al., 2014). Table 1 collects the identities and molecular/fragment ions of some major components present in **M1** and **M2** as identified by comparing LC-MS (negative mode, Figure 1) results with the literature. Both varieties exhibit similar chromatograms, with **M1** having relatively more fraction than **M2**.

**TABLE 1:** LC-MS (negative mode) results of **M1** and **M2**.

S. No	M-H	Mol. Form.	Identification	Ref.
1.	180.1	C_6_H_12_O	β-D-Glucopyranose	Najm et al. (2021)
2.	341.1	C_15_H_18_O_9_	Caffeic acid hexoside	Farag et al. (2014)
3.	179.1	C_16_H_15_O_8_	*O*-Caffeoyl shikimic acid	Otify et al. (2019)
4.	322.0	C_12_H_19_O_10_	Anhydro dihexose	Otify et al. (2019)
5.	425.3	C_17_H_29_O_12_	Acyl sucrose	Najm et al. (2021)
6.	463.1	C_21_H_20_O_12_	Isoquercetin	Farag et al. (2014)
7.	476.0	C_12_H_21_O_12_	Isorhamnetin-3-*O*-glucoside	Najm et al. (2021)
8.	476.0	C_23_H_43_NO_7_P	Sphingolipid conjugate I	Otify et al. (2019)
9.	311.3	C_18_H_31_O_4_	Dihydroxy linolenic acid	Farag et al. (2014)
10.	277.4	C_18_H_29_O_2_	Linolenic acid	Farag et al. (2014)
11.	255.6	C_16_H_31_O_2_	Palmitic acid	Farag et al. (2014)
12.	279.4	C_18_H_31_O_2_	Linoleic acid	Farag et al. (2014)
13.	283.2	C_18_H_36_O_2_	Stearic acid	Eid et al. (2013)
14.	195.0	C_10_H_10_O_4_	ferulic acid	Nematallah et al. (2018)
15.	326.3	C_18_H_32_O_5_	Trihydroxy octadecadienoic acid	Nematallah et al. (2018)
16.	311.4	C_18_H_32_O_4_	Dihydroxy octadecadienoic acid	Nematallah et al. (2018)
17.	594.9	C_27_H_30_O_15_	Luteolin-7-*O*-rutinoside	Nematallah et al. (2018)

GC-MS analysis further indicated the presence of several phytochemical belongings of different classes. For example, quinic acid, oleic acid, trans-13-octadecenoic acid, stearic acid, *O*-caffeoyl shikimic acid, luteolin, trihydroxy-octadecenoic acid, stearic acid linoleic acid, 6-hydroxy 7 methoxy coumarin, 4-hydroxy 6-methylcoumarin and amino acids were tentatively identified (Supplementary Table S3). These components and other metabolites have been well-identified in different varieties of date fruits (Farag et al., 2016; Abdul-Hamid et al., 2019; Perveen and Bokahri, 2020; Souda et al., 2020; Ibrahim et al., 2021).

The FTIR spectrum of the methanolic extracts (Supplementary Figure S1) is also consistent with previous literature (Alam et al., 2022). It has been reported that IR spectrum of dates extracts exhibits multiple peaks responsible for functionalities present in lipid (2,960–2,850 cm^−1^), amide (3,299–3,399 cm^−1^ and 1,591–1,529 cm^−1^ for amine and 1,619–1,691 for carbonyl) and carbohydrates (900–1,200 cm^−1^). As it is clear, the IR spectra of **M1** and **M2** are identical. The spectrum shows a stretching vibrations band at 3,280 cm^−1^ attributed to -OH group, bands at 2,888 and 2,930 cm^−1^ attributed to C_sp3_-H stretching vibration, aromatic C=C stretching vibrations at 1,622 cm^−1^ and C–O deformation vibrations of aliphatic alcohols at 1,009 cm^−1^ (Alam et al., 2022).

### Biological studies

#### Antiproliferative and cytotoxic effect

**FIGURE 2:**
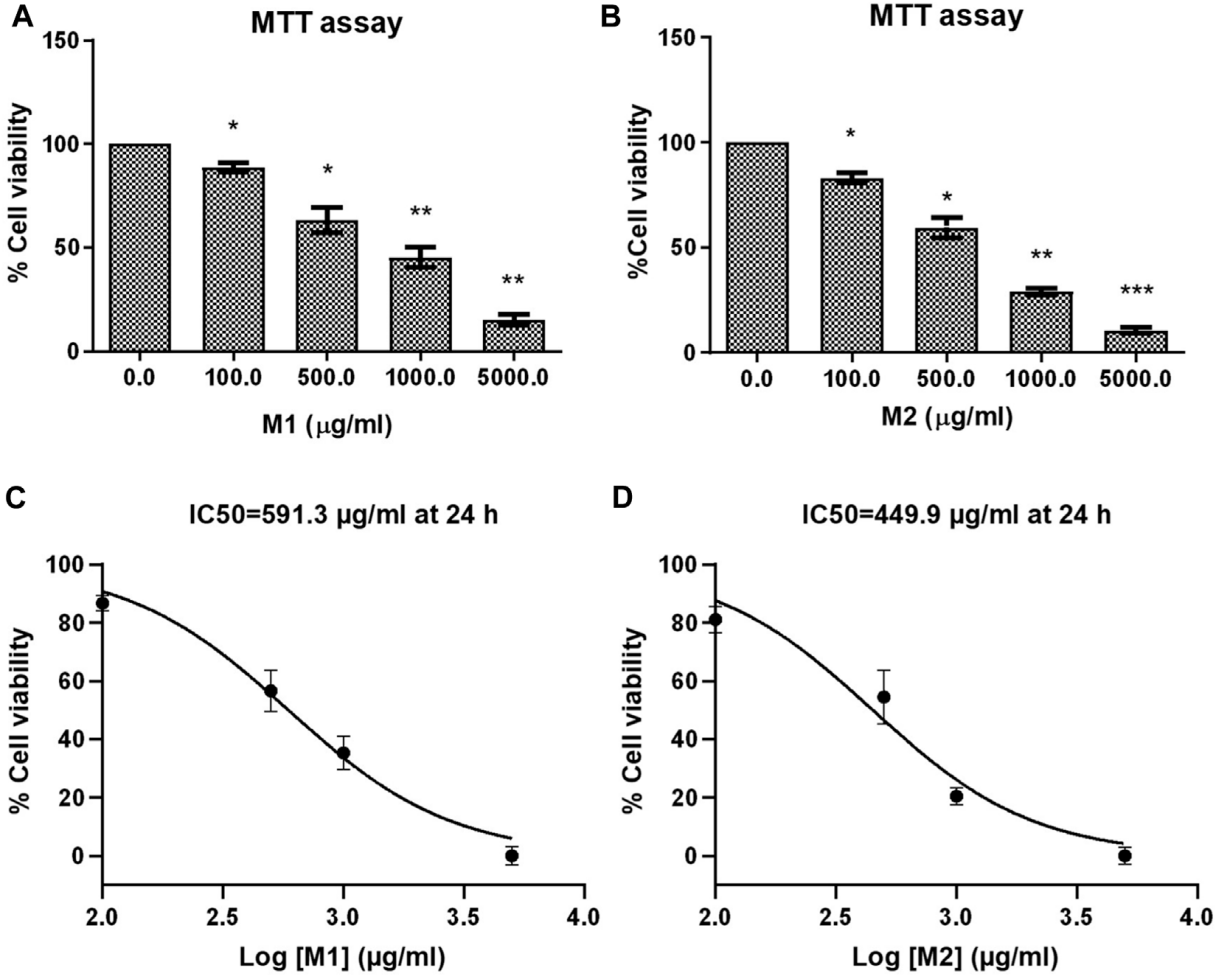
Effect of date extracts M1 and M2 on HCT-116 cells. **(A, B)** Percent (%) cell viability of HCT-116 cells treated with different doses of M1 and M2 (100–5,000 μg/mL) for 24 h. The results shown are the mean ± SEM of three independent experiments performed in triplicate (ns > 0.01, *p < 0.01, **p < 0.001, and ***p < 0.0001 represent significant differences compared with control). **(C, D)** Graph showing IC_50_ of M1 and M2 against HCT-116 colon cancer cell at 24 h.

Using the MTT test, the antiproliferative and cytotoxic effects of the **M1** and **M2** date extract were assessed against colon cancer HCT116 cells for 24 h (Figures 2A, B). The **M1** and **M2** extracts exhibited strong and dose-dependent cytotoxic potential in HCT116 cells. The % cell viability of **M1**-and **M2**-treated HCT116 cells were found to be 88.80% ± 1.33%, 63.26% ± 3.47%, 45.24% ± 2.80%, and 15.28 % ± 1.53%; and 83.07% ± 2.37%, 59.35% ± 4.72%, 28.90% ± 1.49% and 10.63 % ± 1.47% at a dose of 100, 500, 1,000, and 5,000 μg/mL, respectively. IC_50_ values were determined to be 591.3 μg/mL and 449.9 μg/mL for **M1** and **M2**, respectively, revealing the inhibitory potential (Figures 2C, D). Our findings thus demonstrated that both the **M1** and **M2** inhibit colon cancer cell proliferation in a dose-dependent manner.

#### Morphological alterations

**FIGURE 3:**
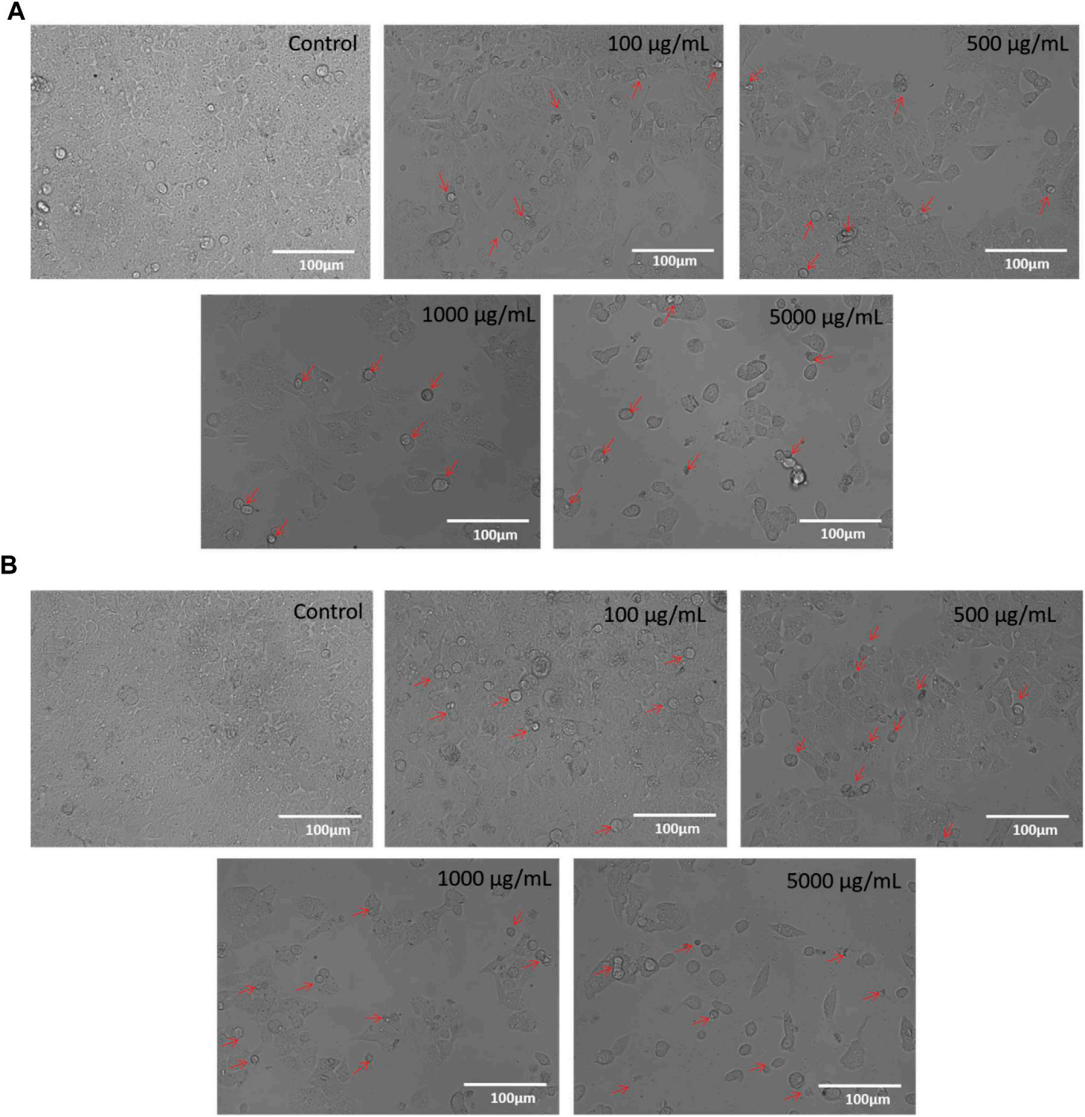
**(A)**: Phase-contrast images of HCT-116 cells treated with either vehicle or different doses of M1 (100–5,000 μg/mL) for 24 h. The photomicrographs shown are the representatives of three independent experiments. **(B)**: Phase-contrast images of HCT-116 cells treated with either vehicle or different doses of M2 (100–5,000 μg/mL) for 24 h. The photomicrographs shown are the representatives of three independent experiments.

Under a phase contrast microscope, the images of control and **M1** & **M2**-treated HCT-116 cells revealed discernible morphological alterations. The control cells showed increased cell growth and intact cell shape. However, in a dose-dependent manner (100, 500, 1,000, and 5,000 μg/mL), significant morphological modifications were observed in the **M1** and **M2**-treated HCT-116 cells (Figures 3A, B). Moreover, **M1** and **M2**-treated HCT-116 cells showed increased detachment and cytoplasmic shrinkage, which led to a rise in the number of floating cells. The findings thus support the hypothesis that treatment with **M1** and **M2** causes cytotoxicity in HCT-116 colon cancer cells.

#### M1 and M2 causes cell death in HCT-116 cells

**FIGURE 4:**
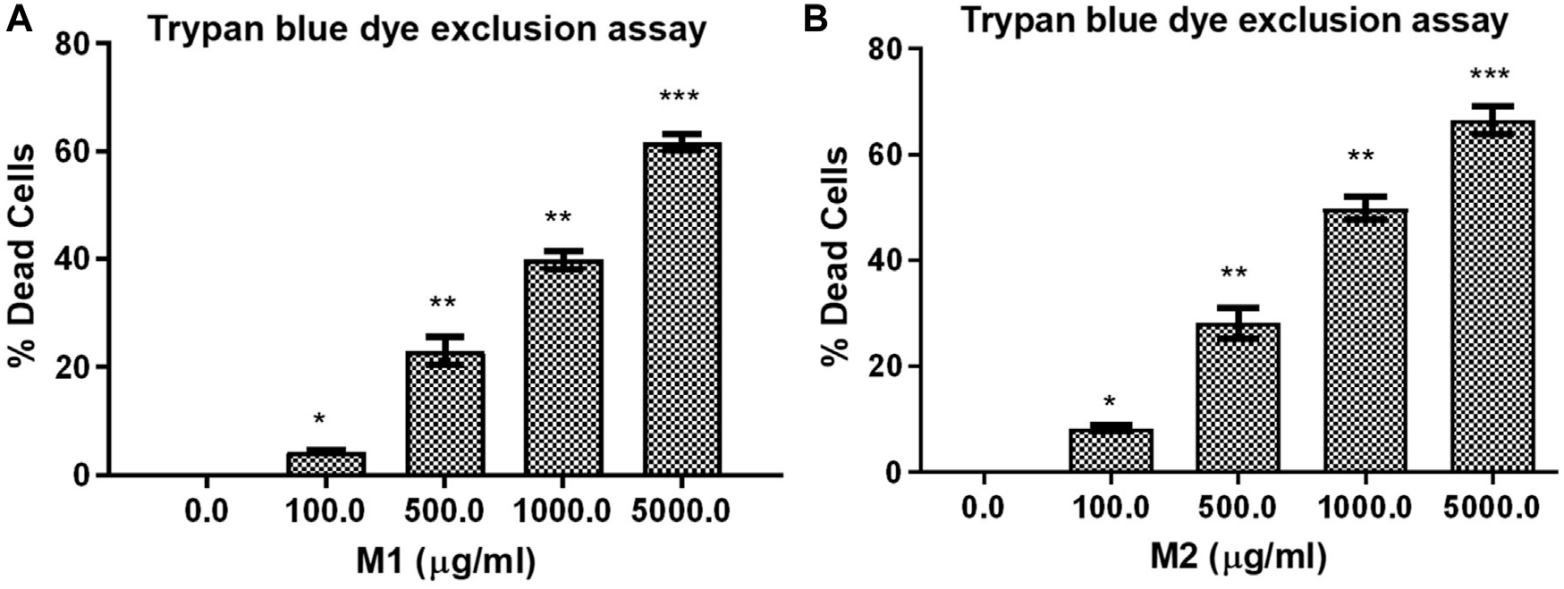
Trypan blue dye exclusion assay. Percent (%) dead cells in HCT-116 cells treated with different doses of **(A)** M1 and **(B)** M2 (100–5,000 μg/mL) for 24 h. The results shown are the mean ± SEM of three independent experiments performed in triplicate (ns > 0.01, *p < 0.01, **p < 0.001, and ***p < 0.0001 represent significant difference compared with control).

Trypan blue dye exclusion assay was used to assess how **M1** and **M2**-treated HCT-116 cells lost viability. Figures 4A, B illustrates the considerable increase in cell mortality in HCT-116 cells after exposure to **M1** and **M2** at various doses (100, 500, 1,000, and 5,000 μg/mL) for 24 h. This result supported the cytotoxic action of **M1** and **M2** on colon cancer cells.

#### Release of cellular LDH in HCT-116 cells

**FIGURE 5:**
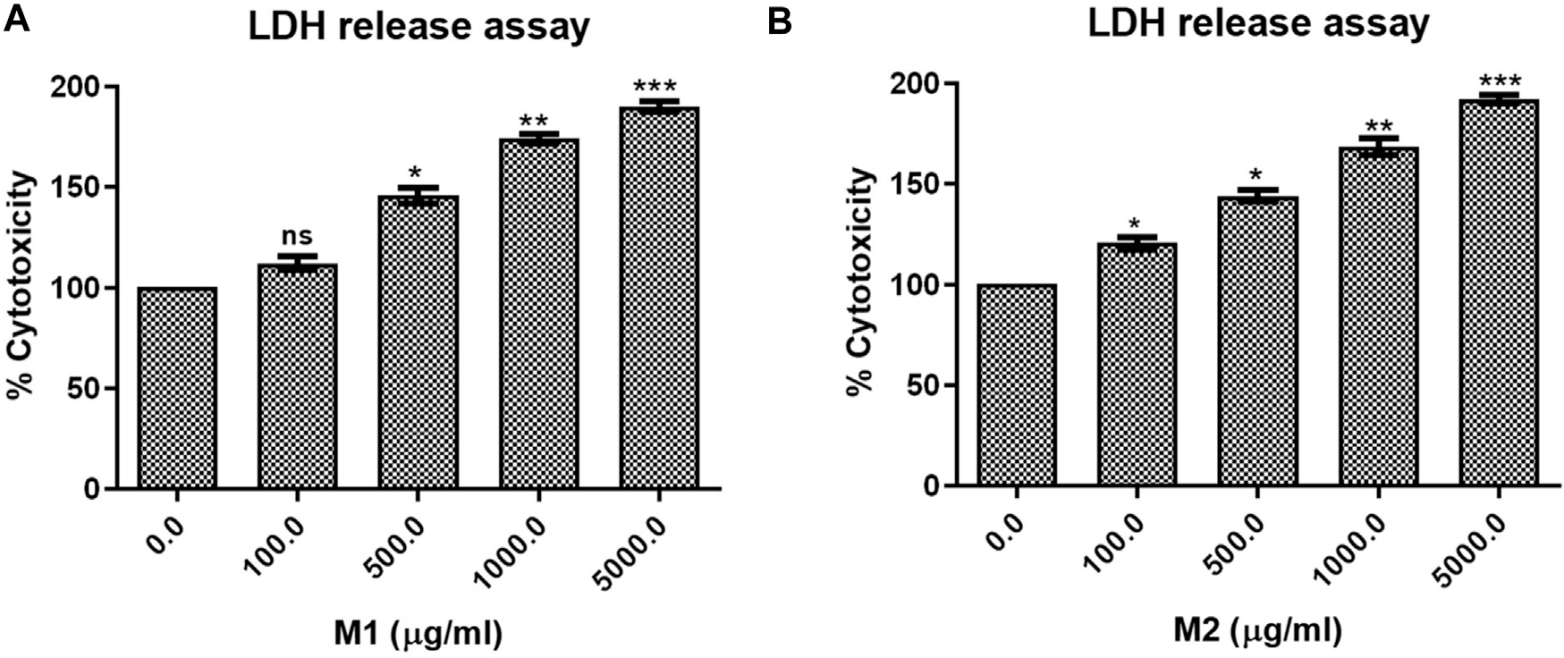
LDH release assay. Percent cytotoxicity in HCT-116 cells treated with different doses of **(A)** M1 and **(B)** M2 (100–5,000 μg/mL) for 24 h. The results shown are the mean ± SEM of three independent experiments performed in triplicate (ns > 0.01, *p < 0.01, **p < 0.001, and ***p < 0.0001 represent significant differences compared to control).

LDH release assay displayed that treatment with both the **M1** and **M2** in HCT-116 cells mediated significant release of LDH, which showed the degree of cellular membrane damage post-treatment. Higher **M1** and **M2** concentrations were found to be significantly more cytotoxic, as evidenced by increased cytotoxicity in HCT-116 cells (Figures 5A, B). The percentage cytotoxicity in **M1**-treated HCT-116 cells, after 24 h of treatment, was found to be 112.08% ± 3.42%, 145.78% ± 3.88%, 174.07% ± 2.25%, and 190.03% ± 2.64% at 100, 500, 1,000, and 5,000 μg/mL dose, respectively. Similarly, after 24 h of treatment with **M2**, HCT-116 cells exhibited percent cytotoxicity of 120.53% ± 3.13%, 144.07% ± 3.00%, 168.46% ± 4.29%, 192.15% ± 1.98% at 100, 500, 1,000, and 5,000 μg/mL dose, respectively. Thus, our results suggest that both the **M1** and **M2** were able to decrease the viability and proliferation in colon cancer cells.

## *In Silico* studies

### Virtual screening

**FIGURE 6:**
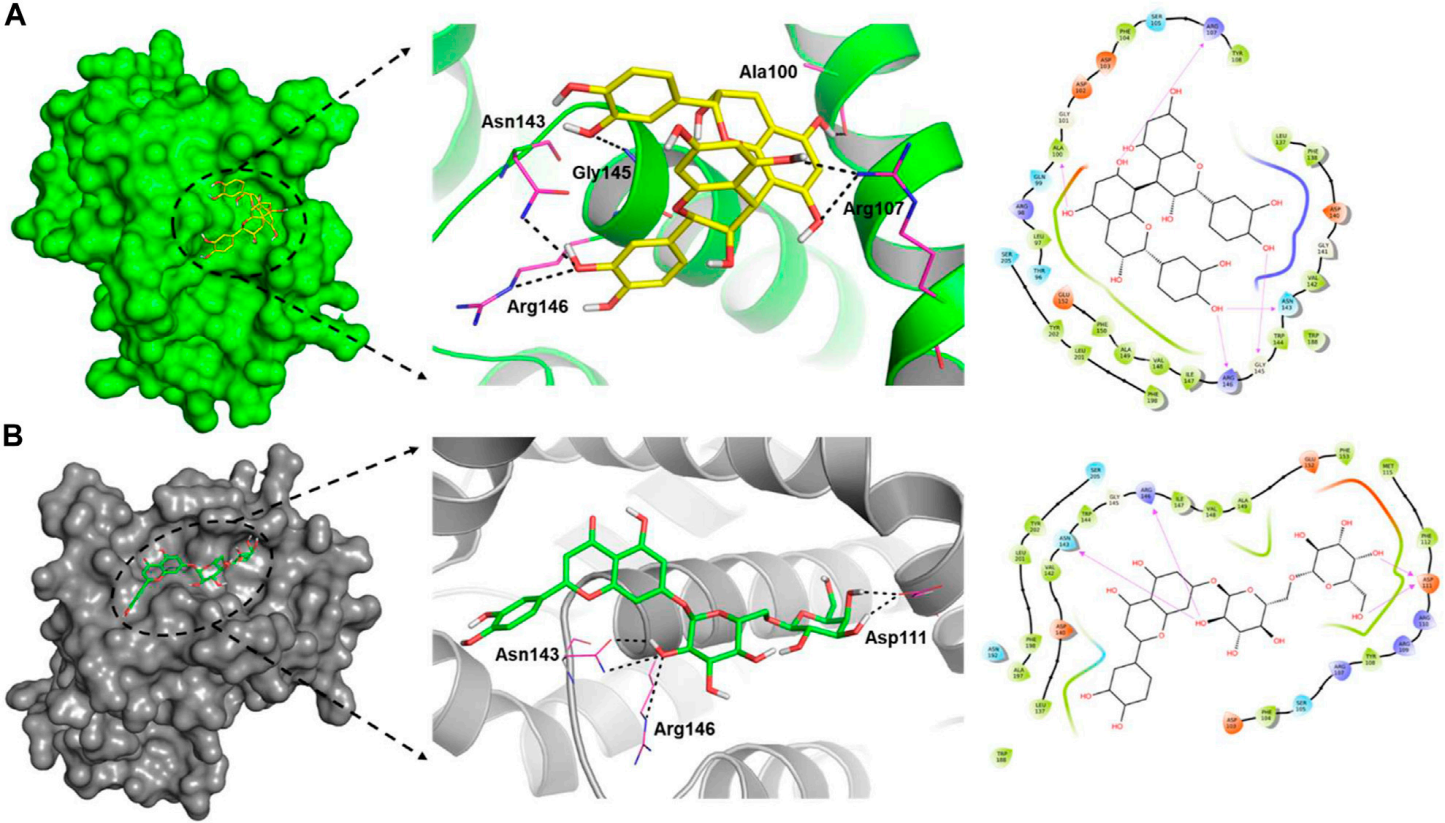
Surface view of the ligand-binding pocket (left), binding poses and interacting fragments (centre) and the H-bonds, hydrophobic interactions, van der Waal’s interactions around 4.0 Ǻ of the binding cavity (right) of procyanidin B2 **(A)** and luteolin-7-*O*-rutinoside **(B)** with Bcl-2 (PDB ID: 5JSN).

It has been demonstrated that the *Phoenix dactylifera* L. extract exhibits anticancer activity by modulating Bcl-2-family proteins (Khan et al., 2021). To identify the critical component(s) responsible for the anticancer activity, we conducted exhaustive *in silico* studies. To this end, ligand-based virtual screening was performed using ninety-four compounds found in *P. dactylifera* against the receptor (PDB: 5JSN). It has been reported that the small molecule may interact with the various receptors of Bcl-2 protein *via* multiple non-covalent interactions (Khosravi et al., 2022; Taghizadeh et al., 2022). Among others, Lys22, Arg26, Asp102, Ser105, Arg106, Arg109, Phe112, Val156, Val159, Asp163, Glu160, and Glu209 which participates in H-bonds and steric interactions (Khosravi et al., 2022). Based on the free binding energies and docking poses, virtual screening of the ligands resulted in procyanidin B2 and luteolin-7-*O*-rutinoside as the most potent candidates (Table 2). As depicted in Figure 6A, procyanidin B2 interacted with various amino acid residues *via* H-bonding (Ala100, Arg107, Asn143, Gly145, and Arg146) and other non-covalent interactions (such as hydrophobic and Van der Waal’s) with a total binding energy of −9.3 kcal/mol. On the other hand, luteolin-7-*O*-rutinoside formed H-bond with Asp111, Asn143 and Arg146 amino acids and yielded a binding energy of −9.1 kcal/mol (Figure 6B). Overall results revealed that the proposed two compounds have an edge over the Bcl-2 complexes attributable to more potent binding abilities.

**TABLE 2:** Ligands name, 3D structures, SMILE format and the virtual screening outputs.

S. No.	Ligand	3D structure	SMILE format	Binding score (kcal/mol)	H-bond residues
1	Procyanidin B2	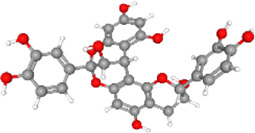	C1C(C(OC2=C1C(=CC3 =C2C4C(C(O3)(OC5= CC(=CC(=C45)O)O)C6=CC(=C(C=C6)O)O)O)O)C7=CC(=C(C=C7)O)O)O	−9.3	Ala100, Arg107, Asn143, Gly145, and Arg146
2	Luteolin-7-*O*-rutinoside	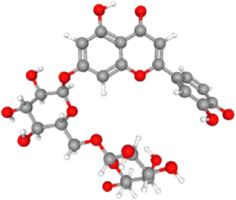	C1=CC(=C(C=C1C2=CC(=O)C3=C(C=C(C=C3O2)OC4C(C(C(C(O4)COC5C(C(C(C(O5)CO)O)O)O)O)O)O)O)O)O	−9.1	Asp111, Asn143 and Arg146

### Molecular dynamics (MD) simulations

To understand the complex stability and interaction profile of the most promising hit compounds inside the active site of Bcl-2, MD simulations of Bcl-2-native, procyanidin B2 and luteolin-7-*O*-rutinoside complexes were performed on a 500 nanosecond (ns) scale. In addition, structural parameters, including RMSD, RMSF, SASA, and Rg were evaluated as a function of time and discussed in the following sub-sections.

### RMS-deviation and RMS-fluctuations

**FIGURE 7:**
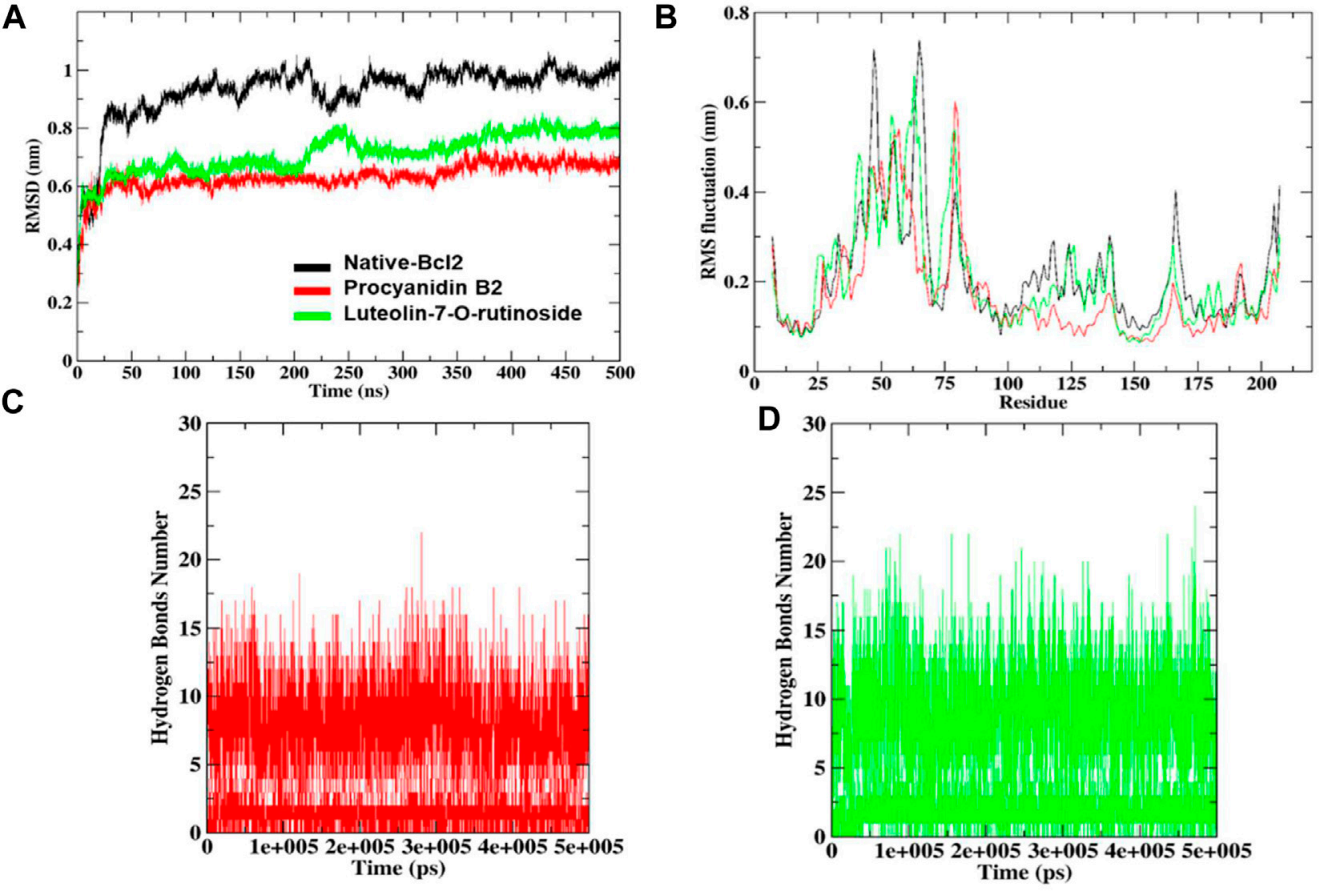
RMSD and RMSF analysis of the complexes of native protein Bcl-2, complexes of shortlisted ligands **(A)**, combined RMS fluctuations **(B)**. The number of hydrogen bonds formed by procyanidin B2 **(C)** and luteolin-7-*O*-rutinoside **(D)**.

The docked complexes were subjected to RMSD analysis to assess the residual flexibility of the Bcl-2 receptor. It was noted that the native protein exhibits higher RMSD fluctuation and reaches equilibrium between 0.8 nm and 1.0 nm. However, in the presence of procyanidin B2, it reached an equilibrium at 0.6 nm and showed steady RMSD (average RMSD value 0.92 nm, Table 3), which remained stable over the 500 ns MD simulation **(** Figure 7A **)**. Similarly, luteolin-7-O-rutinoside and Bcl-2 complexes showed stable equilibrium at 0.6 nm–0.7 nm. Furthermore, they displayed minimal fluctuation over the 500 ns MD simulation. The average RMSD of Luteolin-7-O-rutinoside and Bcl-2 complexes was 0.71 nm. Overall, both procyanidin B2 and luteolin-7-O-rutinoside complexes exhibit stable RMSD values and have a stable binding with Bcl-2 under the given simulation conditions. This also indicates that the studied compounds reached stable and reliable dynamic equilibriums, which bolstered the docking results.

**TABLE 3:** The average RMSD, Rg, and SASA of the native and ligand-protein complexes.

System	RMSD (nm)	Rg (nm)	SASA (nm)^2^
Native	0.92	1.56	111.73
Procyanidin B2	0.63	1.65	112.77
Luteolin-7-*O*-rutinoside	0.71	1.57	115.39

Furthermore, RMSF analysis was implemented to identify the flexible and rigid regions of the complexes and to measure the average atomic flexibility of the Cα-atoms of native Bcl-2 and docked complexes. In the case of native Bcl-2, amino acids residues such as Pro46 ∼0.53 nm, Gly47 ∼ 0.71 nm, Ile48 ∼ 0.64 nm, Arg63 ∼ 0.58 nm, Asp64 ∼ 0.65 nm, Pro65 ∼ 0.73 nm and Val66 ∼ 0.67 nm showed higher fluctuations (Figure 7B). However, fluctuation at 104–112, 162–163 and 201–207 amino acids residue also was found to be higher while other amino acids remain stable. For example, in a complex with procyanidin B2, RMS fluctuations were found in the region Gly79 ∼ 0.60 nm and Ala80 ∼0.57 nm, which is acceptable as these amino acids did not participate in the binding. Similarly, the complex with luteolin-7-O-rutinoside showed RMS-fluctuations at Gly54 ∼0.57 nm, Ala61 ∼0.55 nm and Arg63 ∼0.66 nm values. Overall, the RMSF displayed the highest degree of flexibility, exhibiting stable active site residues interaction compared to the native protein.

### Hydrogen bond monitoring

To underpin the stability of the ligand-protein complex, the number of H-bond was monitored by analyzing the MD trajectories (Figures 7C, D). As can be seen, both compounds procyanidin B2 and luteolin-7-*O*-rutinoside formed 17 and 22 hydrogen bonds, respectively, which increased/remained the same during the 500 ns MD simulation.

### Radius of gyration (Rg) and solvent accessible surface area (SASA)

**FIGURE 8:**
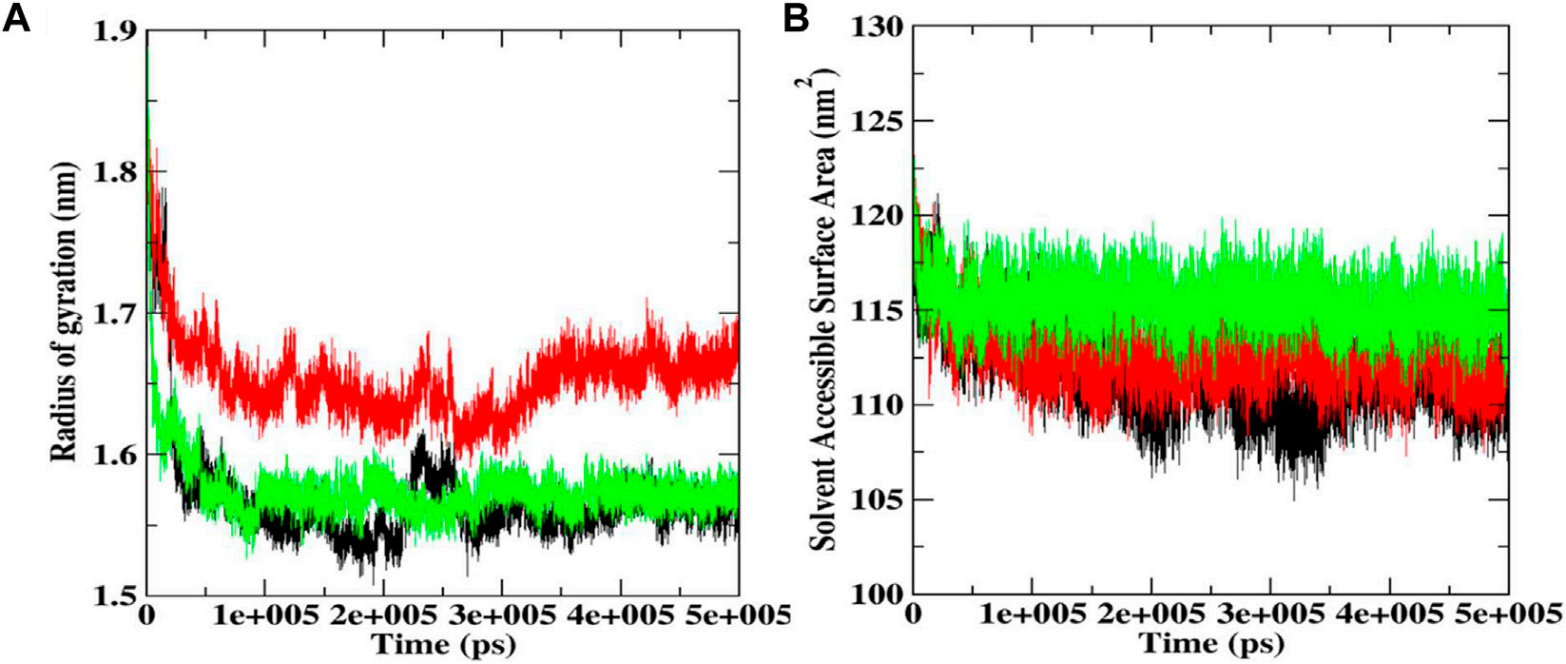
Rg **(A)** and SASA plot **(B)** during 500 ns MD simulations docked complexes of native Bcl-2, and complexes with procyanidin B2 and luteolin-7-*O*-rutinoside.

Rg helps determine protein folding and unfold upon ligand binding, thus giving an idea about the stability of the complex during the simulation. A higher Rg indicates a less compact structure, while a lower Rg means more compactness (Sharma et al., 2022). We found that the average Rg values for the native Bcl-2 protein (1.56 nm) and luteolin-7-O-rutinoside complex (1.57 nm) were almost similar, indicating that the protein will likely maintain a relatively steady value and is stably folded (Figure 8A; Table 2). However, in the case of the procyanidin B2 complex, the average Rg value was 1.65 nm, indicating unfolded structure.

SASA was also conducted to ascertain the interactions between the protein-ligand complex and solvent during the 500 ns MD simulation (Figure 8B; Table 2). It was noted that the average SASA value for the complexes (112.77 and 115.39 nM^2^) of procyanidin B2 and luteolin-7-O-rutinoside, respectively, was better than the native Bcl-2 protein (111.73 nM^2^).

## Discussion

It has been long understood that the phytochemicals found in *Phoenix dactylifera* L. target and inhibit several important biochemical pathways contributing to disease development (Farag et al., 2014; Lamia and Mukti, 2021). The ethnopharmacological significance of *P. dactylifera* L., such as antioxidant, anti-inflammatory anticancer, antimicrobial, etc., is now well established (El Abed et al., 2018). The amount of phytoconstituents, and thus the bioactivity, depends on several factors, including the part of the plant (fruits, seeds, etc.), stage, geographical location, and others. For example, it has been demonstrated that date fruit seeds extract shows anticancer activity against pancreatic (Habib et al., 2014), colorectal (Rezaei et al., 2015), liver (Al-Sheddi, 2019), lung (Al-Sheddi, 2019), and breast (Al-Sheddi, 2019) and other (Al-Zubaidy et al., 2016; Hilary et al., 2021; Habib et al., 2022; Khan et al., 2022) cancer cell lines. On the other hand, Siddiqui et al. (2019) reported that the pulp extract of the *Ajwa* variety exhibit antiproliferative activity against human liver cancer cells (HepG2, IC_50_ = 20.03 and 16.78 mg/mL at 24 and 48 h periods, respectively). Moreover, Khan et al. (2021) demonstrated the apoptosis-inducing potential of *Ajwa* date pulp extract against human triple-negative breast cancer cells (MDA-MB-231, IC_50_ = 17.45 and 16.67 mg/mL at 24 and 48 h, respectively). Khattak et al. (2020) have reported the antiproliferative property of Emirati date fruits extract on human triple-negative breast cancer cell line MDA-MB-231. Besides, the antioxidant and apoptotic potentials of the whole fruit (flesh and pit extracts) is also known (Shahbaz et al., 2022). In addition to the above-mentioned factors, the polarity of extracting solvents also plays an important role; therefore both aqueous and organic solvent systems have been investigated in the past. In a study, it was found that the aqueous extract of a number of date varieties (Saudi Arabian origin) was less bioactive than the methanolic counterparts (Zhang et al., 2017). In a remarkable study, Khan et al. (2016) noted that the methanolic extract of *Ajwa* date fruits exhibit strong anticancer effect on human breast adenocarcinoma (MCF7) (Khan et al., 2016). Besides, other researchers also noted the antitumor activity of methanolic extracts (Mansour et al., 2011; Thouri et al., 2019). Therefore, in the present study, we selected methanol as the solvent to extract date fruits of *Shishi* (**M1**) and *Majdool* (**M2**) cultivars grown in Ha’il region of Saudi Arabia.

As mentioned, (*vide-infra*), the methanolic extract concentrates were subjected to lyophilization and the resulting water-soluble products were used for further studies without any further purification. LC-MS (negative mode) and GC-MS analyses of the extract revealed the presence of various phytochemicals in both varieties. We noted that the chromatograms of **M1** have more peaks than **M2**; therefore, the former has relatively more constituents. Among the main constituents identified were flavonoids, sphingolipids, and fatty acids classes of phytochemicals. Several researchers already report the presence of these constituents in a wide variety of date fruits (see references in the result section). At the same time, we firmly believe the presence of other components escaped the detection. MTT assay of the extracts against colon cancer cells (HCT-116) revealed a dose-dependent inhibitory nature of the compounds (IC_50_ = 591.3 μg/mL and 449.9 μg/mL for **M1** and **M2** at 24, respectively).

It has been demonstrated that the *P. dactylifera* L. extract exhibits anticancer activity by modulating Bcl-2-family proteins which is also expressed in the HCT-116 cell line (El-Far et al., 2021). Considering this, attempts have been made to identify the principal agent(s) present in the extract using computational approaches, including virtual screening and MD studies. Ligand-based virtual screening identified procyanidin B2 and luteolin-7-*O*-rutinoside as the most probable candidates since they could bind with Bcl-2 protein efficiently through various amino acids. MD simulation study further strengthens this observation. Considering the earlier reported values and inhibition mechanism on other cell lines, we believe that the anticancer potential of both *Shishi* and *Majdool* date extracts against colon cancer cells is interesting and requires further biochemical investigation.

## Conclusion

In conclusion, the anticancer activity of methanolic extract of two varieties of dates fruits (*Shishi* **M1** and *Majdool* **M2)** grown in the Ha’il region of Saudi Arabia has been compared. The results of GC-MS and Ft-IR studies indicated the presence of various components in the **M1** and **M2** extracts, which are responsible for dose-dependent cytotoxicity against colon cancer cells (HCT116 cells) through morphological modifications, including cellular membrane damage. The IC_50_ value was 591.3 μg/mL and 449.9 μg/mL for **M1** and **M2**, respectively. Furthermore, Trypan blue dye exclusion assay further supported the cytotoxic action of **M1** and **M2** on colon cancer cells. Extensive virtual screening combined with MD simulations studies at 500 ns revealed that procyanidin B2 and luteolin-7-*O*-rutinoside could be possible agents for the bioactivities. Overall, our data strongly suggest that the consumption of date fruits might prove helpful against colon cancer. Moreover, we also believe that these varieties of date fruits could be utilized as a source of bioactive phytochemicals, leading to the development of Ha’il, Saudi Arabia.

## Data Availability

The original contributions presented in the study are included in the article/Supplementary Material, further inquiries can be directed to the corresponding authors.
