# Supplementary material for: Chemical, biological and in silico assessment of date (P. dactylifera L.) fruits grown in Ha’il region
Source: Front Chem. 2023 Mar 3;11:1138057. doi: 10.3389/fchem.2023.1138057 (PMC10022733; doi:10.3389/fchem.2023.1138057)
Supplement: Supplementary file 1 [file DataSheet1.docx]

**Chemical, biological and in-silico assessment of date (*P. Dactylifera* L.) fruits grown in Ha'il region**

Abdulmohsen Khalaf Dhahi Alsukaibi,^1^ Khalaf M. Alenezi,^1,*^ Ashanul Haque,^1,*^ Irfan Ahmad,^2^ Mohd. Saeed,^3^ Mahima Verma,^4^ Irfan Ahmad Ansari,^4^ Ming-Fa Hsieh^5,*^

^1^ Department of Chemistry, College of Science, University of Ha'il, Kingdom of Saudi Arabia.

^2^ Department of Clinical Laboratory Science, College of Applied Medical Sciences, King Khalid University, Abha, Kingdom of Saudi Arabia.

^3^ Department of Biology, College of Science, University of Ha'il, Kingdom of Saudi Arabia.

^4^ Department of Biosciences, Integral University, Lucknow, 226026, India.

^5^ Department of Biomedical Engineering, Chung Yuan Christian University, 200 Chung Pei Road, Chung Li District, Taoyuan City, Taiwan 32023, Taiwan

E-mail: [a.haque@uoh.edu.sa](mailto:a.haque@uoh.edu.sa) (AH); [k.alenezi@uoh.edu.sa](mailto:k.alenezi@uoh.edu.sa); [mfhsieh@cycu.edu.tw](mailto:mfhsieh@cycu.edu.tw) (MFH)

**Supporting information**

**Figure S1**: IR (ATR) spectra of date fruits extracts.


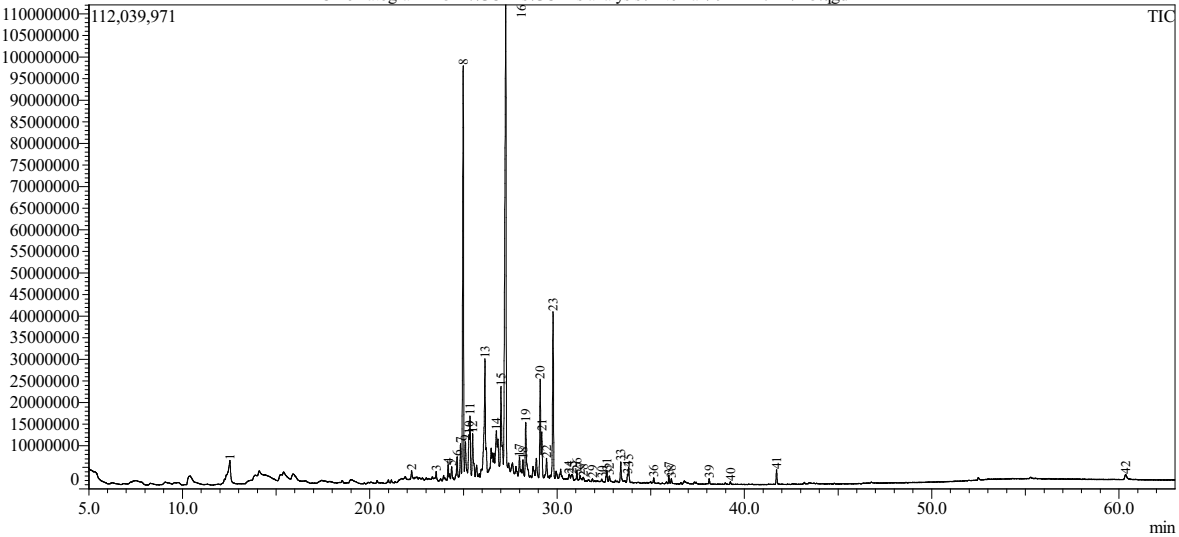


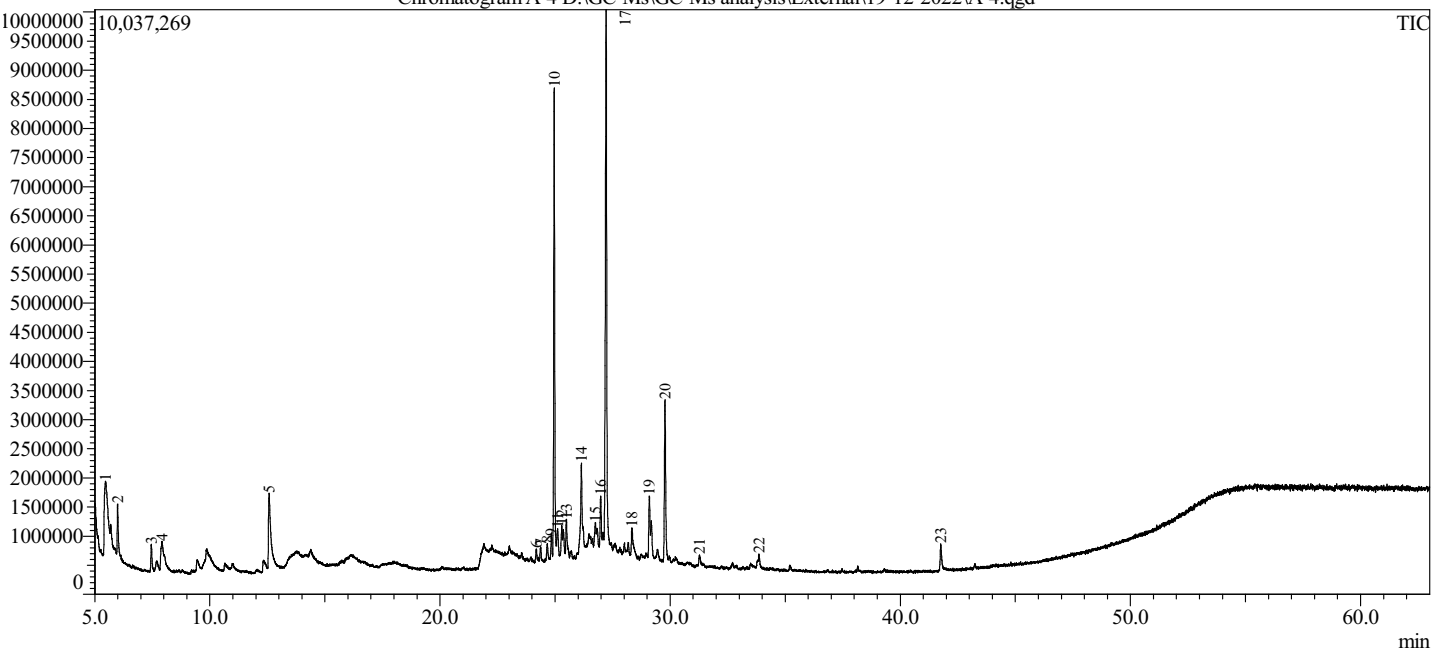


**Figure S2**: Gas chromatography–mass spectrometry (GC-MS) analysis of **M1** (top) and **M2** (bottom). Peaks of major compounds were revealed with different percentage of intensity and retention time (RT).

**Table T1**: Sample, local name, amount used for extraction (in 1:3 w/v MeOH at RT) and solid obtained after lyophilization.

| **Sample Code** | **Local/ethnic name** | **Pieces**  **(Weight, gm)** | **Sample** | **Solid**  **obtained (gm)** |
| --- | --- | --- | --- | --- |
| **M1** | *Shishi* | 3  (36.11) | 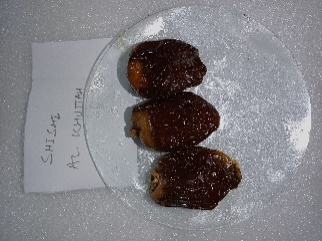 | 9.1 |
| **M2** | *Majdool* | 3  (46.22) | 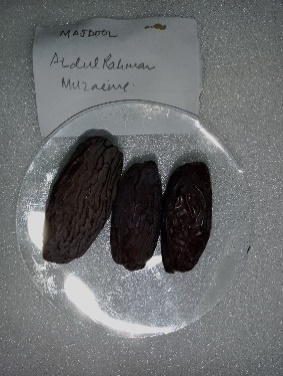 | 15 |

**Table T2**: Types and names of constituents used for the *in-silico* evaluation. Pubmed link for the studied compounds are also give.

| **S. No.** | **Class & Constituents** | **PubMed link/Chart** |
| --- | --- | --- |
| **Carotenoids** | | |
|  | Antheraxanthin | [Antheraxanthin \| C40H56O3 - PubChem (nih.gov)](https://pubchem.ncbi.nlm.nih.gov/compound/5281223#section=2D-Structure) |
|  | Cryptoxanthine | [beta-Cryptoxanthin \| C40H56O - PubChem (nih.gov)](https://pubchem.ncbi.nlm.nih.gov/compound/5281235#section=2D-Structure) |
|  | Echinenone | [Echinenone \| C40H54O - PubChem (nih.gov)](https://pubchem.ncbi.nlm.nih.gov/compound/5281236) |
|  | Flavoxanthin | [Flavoxanthin \| C40H56O3 - PubChem (nih.gov)](https://pubchem.ncbi.nlm.nih.gov/compound/5281238#section=2D-Structure) |
|  | Lutein | [Lutein \| C40H56O2 - PubChem (nih.gov)](https://pubchem.ncbi.nlm.nih.gov/compound/5281243#section=2D-Structure) |
|  | Lycopene | [Lycopene \| C40H56 - PubChem (nih.gov)](https://pubchem.ncbi.nlm.nih.gov/compound/446925) |
|  | Neoxanthin | [(3S,3'S,5R,5'R,6S,6'R,9'Z)-5,6-Epoxy-6',7'-didehydro-5,6,5',6'-tetrahydro-beta,beta-carotene-3,3',5'-triol \| C40H56O4 - PubChem (nih.gov)](https://pubchem.ncbi.nlm.nih.gov/compound/12358959) |
|  | Violaxanthin | [Violaxanthin \| C40H56O4 - PubChem (nih.gov)](https://pubchem.ncbi.nlm.nih.gov/compound/448438#section=2D-Structure) |
|  | α-carotenes | [alpha-Carotene \| C40H56 - PubChem (nih.gov)](https://pubchem.ncbi.nlm.nih.gov/compound/6419725) |
|  | β-carotene | [SID 319295400 - PubChem (nih.gov)](https://pubchem.ncbi.nlm.nih.gov/substance/319295400) |
|  | β-cryptoxanthin | [beta-Cryptoxanthin \| C40H56O - PubChem (nih.gov)](https://pubchem.ncbi.nlm.nih.gov/compound/5281235) |
|  | ϒ-carotenes | [gamma-Carotene \| C40H56 - PubChem (nih.gov)](https://pubchem.ncbi.nlm.nih.gov/compound/5280791) |
|  | Zeaxanthin | [Zeaxanthin \| C40H56O2 - PubChem (nih.gov)](https://pubchem.ncbi.nlm.nih.gov/compound/5280899) |
| **Phenolic acids** | | |
|  | 3-Hydroxybenzoic acid | [3-Hydroxybenzoic acid \| C7H6O3 - PubChem (nih.gov)](https://pubchem.ncbi.nlm.nih.gov/compound/7420#section=3D-Conformer) |
|  | Caffeic Acid | [Caffeic acid \| C9H8O4 - PubChem (nih.gov)](https://pubchem.ncbi.nlm.nih.gov/compound/689043) |
|  | Cathechin | [Cianidanol \| C15H14O6 - PubChem (nih.gov)](https://pubchem.ncbi.nlm.nih.gov/compound/9064#section=2D-Structure) |
|  | Chlorogenic Acid/3-Caffeoylquinic Acid | [Chlorogenic acid \| C16H18O9 - PubChem (nih.gov)](https://pubchem.ncbi.nlm.nih.gov/compound/1794427) |
|  | Cinnamic Acid | [Cinnamic acid \| C9H8O2 - PubChem (nih.gov)](https://pubchem.ncbi.nlm.nih.gov/compound/444539) |
|  | Ellagic Acid | [Ellagic acid \| C14H6O8 - PubChem (nih.gov)](https://pubchem.ncbi.nlm.nih.gov/compound/5281855) |
|  | Esculetin | [Esculetin \| C9H6O4 - PubChem (nih.gov)](https://pubchem.ncbi.nlm.nih.gov/compound/5281416) |
|  | Ferulic Acid | [Ferulic acid \| C10H10O4 - PubChem (nih.gov)](https://pubchem.ncbi.nlm.nih.gov/compound/Ferulic-acid#section=3D-Conformer) |
|  | Gallic Acid | [Gallic acid \| C7H6O5 - PubChem (nih.gov)](https://pubchem.ncbi.nlm.nih.gov/compound/370) |
|  | Hydroxyphenylacetic Acid | [4-Hydroxyphenylacetic acid \| C8H8O3 - PubChem (nih.gov)](https://pubchem.ncbi.nlm.nih.gov/compound/127) |
|  | Isovanillic Acid | [3-Hydroxy-4-methoxybenzoic acid \| C8H8O4 - PubChem (nih.gov)](https://pubchem.ncbi.nlm.nih.gov/compound/12575#section=3D-Conformer) |
|  | Itaconic Acid | [Itaconic acid \| C5H6O4 - PubChem (nih.gov)](https://pubchem.ncbi.nlm.nih.gov/compound/811) |
|  | m-coumaric acid | [3-Hydroxycinnamic acid \| C9H8O3 - PubChem (nih.gov)](https://pubchem.ncbi.nlm.nih.gov/compound/637541#section=3D-Conformer) |
|  | o-cinnamic acid | *See chart* |
|  | o–coumaric acid | [2-Hydroxycinnamic acid \| C9H8O3 - PubChem (nih.gov)](https://pubchem.ncbi.nlm.nih.gov/compound/637540#section=3D-Conformer) |
|  | 5-o-caffeoylshikimic acid | [5-O-Caffeoylshikimic acid \| C16H16O8 - PubChem (nih.gov)](https://pubchem.ncbi.nlm.nih.gov/compound/5281762#section=2D-Structure) |
|  | p-coumaric acid | [4-Hydroxycinnamic acid \| C9H8O3 - PubChem (nih.gov)](https://pubchem.ncbi.nlm.nih.gov/compound/637542) |
|  | Pelargonin | [Pelargonin \| C27H31O15+ - PubChem (nih.gov)](https://pubchem.ncbi.nlm.nih.gov/compound/441772#section=3D-Conformer) |
|  | Phenylacetic Acid | [Phenylacetic acid \| C8H8O2 - PubChem (nih.gov)](https://pubchem.ncbi.nlm.nih.gov/compound/999) |
|  | P-Hydroxybenzoic Acid | [4-Hydroxybenzoic acid \| C7H6O3 - PubChem (nih.gov)](https://pubchem.ncbi.nlm.nih.gov/compound/135#section=3D-Conformer) |
|  | Protocatechuic Acid | [3,4-Dihydroxybenzoic acid \| C7H6O4 - PubChem (nih.gov)](https://pubchem.ncbi.nlm.nih.gov/compound/72#section=3D-Conformer) |
|  | Sinapic Acid | [Sinapic acid \| C11H12O5 - PubChem (nih.gov)](https://pubchem.ncbi.nlm.nih.gov/compound/637775#section=3D-Conformer) |
|  | Syringic Acid | [Syringic acid \| C9H10O5 - PubChem (nih.gov)](https://pubchem.ncbi.nlm.nih.gov/compound/10742#section=3D-Conformer) |
|  | Tannic Acid | [Tannic acid \| C76H52O46 - PubChem (nih.gov)](https://pubchem.ncbi.nlm.nih.gov/compound/16129778#section=2D-Structure) |
|  | Tyrosol | [2-(4-Hydroxyphenyl)ethanol \| C8H10O2 - PubChem (nih.gov)](https://pubchem.ncbi.nlm.nih.gov/compound/10393) |
|  | Vanillic Acid | [Vanillic acid \| C8H8O4 - PubChem (nih.gov)](https://pubchem.ncbi.nlm.nih.gov/compound/8468#section=3D-Conformer) |
|  | Chrysoeriol | [Chrysoeriol \| C16H12O6 - PubChem (nih.gov)](https://pubchem.ncbi.nlm.nih.gov/compound/5280666) |
|  | 4-Caffeoylshikimic acid | [4-o-Caffeoylshikimic acid \| C16H16O8 - PubChem (nih.gov)](https://pubchem.ncbi.nlm.nih.gov/compound/49821869) |
|  | 5-Caffeoylshikimic acid | [5-O-Caffeoylshikimic acid \| C16H16O8 - PubChem (nih.gov)](https://pubchem.ncbi.nlm.nih.gov/compound/5281762) |
|  | 3-Caffeoylshikimic acid | [3-Caffeoylshikimic acid \| C16H16O8 - PubChem (nih.gov)](https://pubchem.ncbi.nlm.nih.gov/compound/10131826) |
|  | Salicylic acid | [Salicylic acid \| HOC6H4COOH - PubChem (nih.gov)](https://pubchem.ncbi.nlm.nih.gov/compound/338) |
|  | Curcumin | [Curcumin \| C21H20O6 - PubChem (nih.gov)](https://pubchem.ncbi.nlm.nih.gov/compound/969516) |
|  | Isorhamnetin | [Isorhamnetin \| C16H12O7 - PubChem (nih.gov)](https://pubchem.ncbi.nlm.nih.gov/compound/5281654) |
|  | Resorcinol | [Resorcinol \| C6H6O2 - PubChem (nih.gov)](https://pubchem.ncbi.nlm.nih.gov/compound/5054) |
| **Flavonoids** | | |
|  | Apigenin | [Apigenin \| C15H10O5 - PubChem (nih.gov)](https://pubchem.ncbi.nlm.nih.gov/compound/5280443#section=3D-Conformer) |
|  | Diosmetin | [Diosmetin \| C16H12O6 - PubChem (nih.gov)](https://pubchem.ncbi.nlm.nih.gov/compound/5281612#section=3D-Conformer) |
|  | Flavonol glycoside | [Flavonol 3-O-D-glucoside \| C21H20O8 - PubChem (nih.gov)](https://pubchem.ncbi.nlm.nih.gov/compound/11953828) |
|  | Isoquerectin | [Isoquercitrin \| C21H20O12 - PubChem (nih.gov)](https://pubchem.ncbi.nlm.nih.gov/compound/Isoquercitrin#section=3D-Conformer) |
|  | Kempferol | [Kaempferol \| C15H10O6 - PubChem (nih.gov)](https://pubchem.ncbi.nlm.nih.gov/compound/5280863#section=3D-Conformer) |
|  | Luteolin | [Luteolin \| C15H10O6 - PubChem (nih.gov)](https://pubchem.ncbi.nlm.nih.gov/compound/5280445#section=3D-Conformer) |
|  | Procyanidin | [Procyanidin \| C30H26O13 - PubChem (nih.gov)](https://pubchem.ncbi.nlm.nih.gov/compound/107876#section=3D-Conformer) |
|  | Quercetin | [Quercetin \| C15H10O7 - PubChem (nih.gov)](https://pubchem.ncbi.nlm.nih.gov/compound/5280343#section=3D-Conformer) |
|  | Rutin | [Rutin \| C27H30O16 - PubChem (nih.gov)](https://pubchem.ncbi.nlm.nih.gov/compound/5280805#section=3D-Conformer) |
|  | Procyanidin B1 | [Procyanidin B1 \| C30H26O12 - PubChem (nih.gov)](https://pubchem.ncbi.nlm.nih.gov/compound/11250133) |
|  | Procyanidin B2 | [Procyanidin B2 \| C30H26O12 - PubChem (nih.gov)](https://pubchem.ncbi.nlm.nih.gov/compound/122738) |
|  | Procyanidin A2 | [Proanthocyanidin A2 \| C30H24O12 - PubChem (nih.gov)](https://pubchem.ncbi.nlm.nih.gov/compound/124025) |
|  | Kaempferol hexoside | [Astragalin \| C21H20O11 - PubChem (nih.gov)](https://pubchem.ncbi.nlm.nih.gov/compound/5282102#section=2D-Structure) |
|  | Luteolin-7-O-β-D–glucoside | [SID 12015660 - PubChem (nih.gov)](https://pubchem.ncbi.nlm.nih.gov/substance/12015660) |
|  | Isorhamnetin-3-O-glucoside | [isorhamnetin-3-O-glucoside \| C22H22O12 - PubChem (nih.gov)](https://pubchem.ncbi.nlm.nih.gov/compound/5318645) |
|  | Naringin | [Naringin \| C27H32O14 - PubChem (nih.gov)](https://pubchem.ncbi.nlm.nih.gov/compound/442428) |
|  | Myricetin | [Myricetin \| C15H10O8 - PubChem (nih.gov)](https://pubchem.ncbi.nlm.nih.gov/compound/5281672) |
|  | Morin | [Morin \| C15H10O7 - PubChem (nih.gov)](https://pubchem.ncbi.nlm.nih.gov/compound/5281670) |
|  | Galangin | [Galangin \| C15H10O5 - PubChem (nih.gov)](https://pubchem.ncbi.nlm.nih.gov/compound/5281616) |
|  | Luteolin-7-O-rutinoside | [luteolin-7-O-rutinoside \| C27H30O16 - PubChem (nih.gov)](https://pubchem.ncbi.nlm.nih.gov/compound/14032966) |
|  | Quercetin3-O-rutinoside | [Rutin \| C27H30O16 - PubChem (nih.gov)](https://pubchem.ncbi.nlm.nih.gov/compound/5280805) |
|  | Isorhamnetin-3-O-rutinoside | [Narcissoside \| C28H32O16 - PubChem (nih.gov)](https://pubchem.ncbi.nlm.nih.gov/compound/5481663) |
|  | kaempferol-3-glucoside | [Astragalin \| C21H20O11 - PubChem (nih.gov)](https://pubchem.ncbi.nlm.nih.gov/compound/5282102) |
|  | glucopyranoside | [D-Glucose \| C6H12O6 - PubChem (nih.gov)](https://pubchem.ncbi.nlm.nih.gov/compound/5793) |
|  | Isoquercitrin | [Isoquercitrin \| C21H20O12 - PubChem (nih.gov)](https://pubchem.ncbi.nlm.nih.gov/compound/5280804) |
|  | Daidzein | [Daidzein \| C15H10O4 - PubChem (nih.gov)](https://pubchem.ncbi.nlm.nih.gov/compound/5281708) |
|  | Genistein | [Genistein \| C15H10O5 - PubChem (nih.gov)](https://pubchem.ncbi.nlm.nih.gov/compound/5280961) |
|  | Glycitein | [Glycitein \| C16H12O5 - PubChem (nih.gov)](https://pubchem.ncbi.nlm.nih.gov/compound/5317750) |
|  | Formononetin | [Formononetin \| C16H12O4 - PubChem (nih.gov)](https://pubchem.ncbi.nlm.nih.gov/compound/5280378) |
|  | Petunidin | [Petunidin \| C16H13O7+ - PubChem (nih.gov)](https://pubchem.ncbi.nlm.nih.gov/compound/441774) |
|  | **Tocopherol, Tocotrienol, & Phytoestrogens** | |
|  | campesterol | [Campesterol \| C28H48O - PubChem (nih.gov)](https://pubchem.ncbi.nlm.nih.gov/compound/173183) |
|  | cholesterol | [Cholesterol \| C27H46O - PubChem (nih.gov)](https://pubchem.ncbi.nlm.nih.gov/compound/5997) |
|  | tocotrienol | [Tocotrienol \| C26H38O2 - PubChem (nih.gov)](https://pubchem.ncbi.nlm.nih.gov/compound/9929901) |
|  |  |  |
|  | ϒ-tocopherol | [gamma-Tocopherol \| C28H48O2 - PubChem (nih.gov)](https://pubchem.ncbi.nlm.nih.gov/compound/92729) |
|  | α- Tocopherol | [SID 57393415 - PubChem (nih.gov)](https://pubchem.ncbi.nlm.nih.gov/substance/57393415) |
|  | α-Tocotrienol | [alpha-Tocotrienol \| C29H44O2 - PubChem (nih.gov)](https://pubchem.ncbi.nlm.nih.gov/compound/5282347) |
|  | β-Sitosterol | [beta-Sitosterol \| C29H50O - PubChem (nih.gov)](https://pubchem.ncbi.nlm.nih.gov/compound/222284) |
|  | Δ5,24-stigmastadienol | [5,24-Stigmastadienol \| C29H48O - PubChem (nih.gov)](https://pubchem.ncbi.nlm.nih.gov/compound/70130285) |
|  | Δ5-avenasterol | [Fucosterol \| C29H48O - PubChem (nih.gov)](https://pubchem.ncbi.nlm.nih.gov/compound/5281326) |
|  | Δ7-avenasterol | [delta7-Avenasterol \| C29H48O - PubChem (nih.gov)](https://pubchem.ncbi.nlm.nih.gov/compound/12795736) |
|  | Δ7-stigmastenol | [SID 319287569 - PubChem (nih.gov)](https://pubchem.ncbi.nlm.nih.gov/substance/319287569) |
|  | Matairesinol | [Matairesinol \| C20H22O6 - PubChem (nih.gov)](https://pubchem.ncbi.nlm.nih.gov/compound/119205) |
|  | Lariciresinol | [Lariciresinol \| C20H24O6 - PubChem (nih.gov)](https://pubchem.ncbi.nlm.nih.gov/compound/332427) |
|  | Secoisolariciresinol | [Secoisolariciresinol \| C20H26O6 - PubChem (nih.gov)](https://pubchem.ncbi.nlm.nih.gov/compound/65373) |
|  | Pinoresinol | [Pinoresinol \| C20H22O6 - PubChem (nih.gov)](https://pubchem.ncbi.nlm.nih.gov/compound/73399) |
|  | Coumestrol | [Coumestrol \| C15H8O5 - PubChem (nih.gov)](https://pubchem.ncbi.nlm.nih.gov/compound/5281707) |

**Table T3**. Tentative identification of metabolites in methanolic extract of P. dactylifera fruits using GC-MS technique.

| **Compound** | **Ref** |
| --- | --- |
| Quinic acid | Biomass Conversion and Biorefinery (2022) 12:3771–3781 |
| Oleic acid | Biomass Conversion and Biorefinery (2022) 12:3771–3781 |
| Trans-13-Octadecenoic acid | Biomass Conversion and Biorefinery (2022) 12:3771–3781 |
| Stearic acid | 10.1039/C5FO01570G |
| O-Caffeoyl shikimic acid | 10.1039/C5FO01570G |
| Luteolin | 10.1039/C5FO01570G |
| Trihydroxy-octadecenoic acid | 10.1039/C5FO01570G |
| Aspartic acid | doi:10.1111/ijfs.14783 |
| Formic Acid Hydrazide | https://doi.org/10.1016/j.sjbs.2019.11.029 |
| Linoleic acid | <https://doi.org/10.1016/j.phytol.2019.03.004> |
| 6-hydroxy 7 methoxy coumarin | <https://doi.org/10.1016/j.phytol.2019.03.004> |
| 4-Hydroxy- 6 methylcoumarin | <https://doi.org/10.1016/j.phytol.2019.03.004> |
